# Gastric Aspergillosis in a COVID-19 Patient

**DOI:** 10.7759/cureus.34897

**Published:** 2023-02-12

**Authors:** Valeria Hinojosa, Sabina Cesena De la Fuente, Najia Hussaini, Joseph Varon

**Affiliations:** 1 Research, Universidad Autónoma de Baja California, Tijuana, MEX; 2 Research, Caribbean Medical University, Houston, USA; 3 Critical Care Medicine, United Memorial Medical Center, Houston, USA

**Keywords:** gastric ulceration, gastrointestinal bleeding, coronavirus disease, aspergillus, pneumonia, covid-19

## Abstract

The SARS-CoV-2 virus, which causes Coronavirus-19 infection (COVID-19), frequently elicits the development of depressed immunity, therefore, opportunistic infections. Opportunistic organisms are commonly present in the human body without causing critical illness. However, they can also lead to pathologic illness when a person is immunocompromised. Aspergillosis is among the many opportunistic infections. Even though this infection primarily involves the respiratory system and is less likely to be found in the gastrointestinal tract, we report a case of a COVID-19 individual that developed massive gastrointestinal bleeding whose condition deteriorated, and the pathological examination revealed gastric aspergillosis. Although not common, gastric aspergillosis should be considered while treating patients with COVID-19 who present gastrointestinal symptoms.

## Introduction

As of September 2021, the world has seen more than 600 million cases of coronavirus 2019 infection caused by the SARS-CoV-2. This illness can present mild to severe symptoms and, in worst-case scenarios, patients may require assisted mechanical ventilation [1]. In many cases of COVID-19, co-infections occur due to secondary immunosuppression [2]. These infections are attributable to the infection of CD4 and CD8 T lymphocytes [3].

Aspergillosis is an opportunistic infection that can be seen in patients with severe immunosuppression, such as in cases of severe COVID-19. The organs most commonly affected by this infectious illness are the lungs. However, as an invasive disease, it can be disseminated to other organs [4]. We recently had one such case.

## Case presentation

A 51-year-old Caucasian woman presented on September 2021 to our hospital with complaints of shortness of breath, cough, nasal congestion, diffuse body aches, fever and chills for three weeks prior to admission. She had not been vaccinated against COVID-19. She has a past medical history significant for thyroid malignancy which was resected, and is in complete remission, fibromyalgia and well-controlled chronic asthma with albuterol.

**Figure 1:**
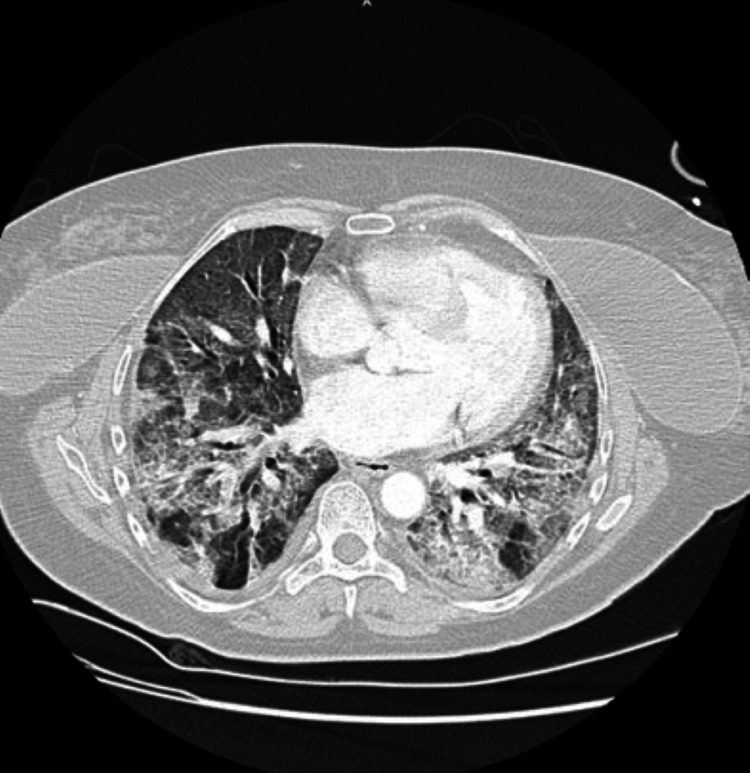
CT of the chest with IV contrast depicting ground glass opacities consistent with a COVID-19 infection.

On initial physical examination, her blood pressure was 114/77 mmHg, heart rate 93/min, respiratory rate 38/min, SpO2 89% while breathing supplemental oxygen with mask reservoir at 15 L/min, temperature 37.2°C. A computed tomography (CT) scan of the chest depicted bilateral ground glass pulmonary opacities, among consolidation, septal thickening, and minimal pleural effusion (See Figure 1).

Her Ichikado score was calculated at 220 [5]. Her RT-PCR nasopharyngeal swab was positive for SARS-CoV-2. She was admitted to the hospital, and was started on intravenous methylprednisolone 1g Q24H for three days, then the dose was progressively reduced, ascorbic acid, thiamine, oral atorvastatin, zinc and melatonin. In addition, she received low molecular weight heparin at full therapeutic doses.

**Figure 2:**
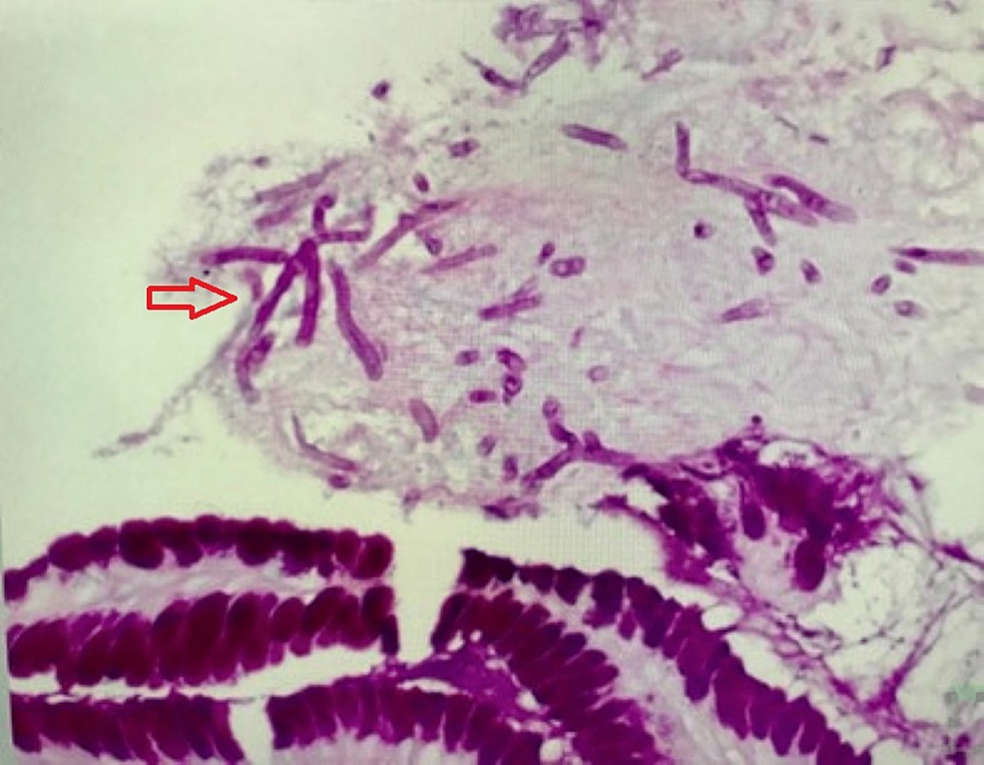
Periodic acid-Schiff highlighted the presence of aggregates of fungal organisms that correlate to Aspergillus spp.

**Figure 3:**
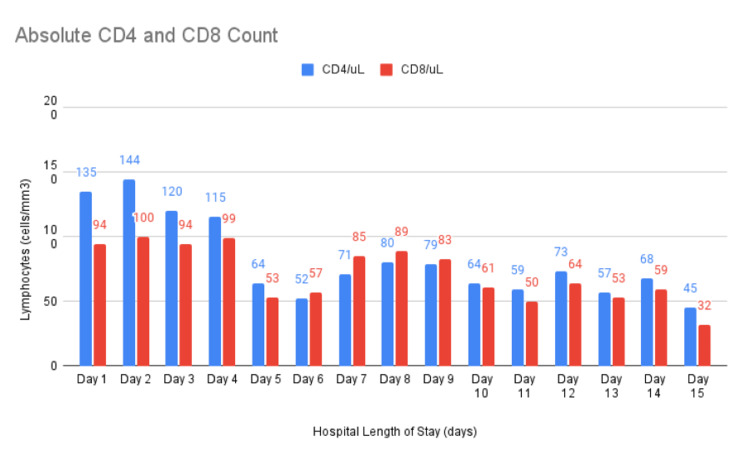
This graph demonstrates the decline in absolute CD4+ and CD8+ T-Lymphocytes levels, expressed in cells/mm3, throughout the patient’s hospital stay.

Supplemental oxygen given by a high-flow nasal cannula (HFNC) parameter was in the range of 30-40 l/min and FiO2 80-100%. After two weeks, the patient was managed with bilevel positive airway pressure (BiPAP) with FiO2 ranging from 80-100%. Despite aggressive management, her condition remained unstable, and unchanged for another week. At that time she developed worsening respiratory distress and increased work of breathing that required invasive assisted mechanical ventilation as well as initiating a vasopressor. The patient developed acute hypovolemic shock after one week of mechanical ventilation due to severe anemia, requiring three blood transfusions. An upper gastrointestinal endoscopy was performed to rule out gastrointestinal tract bleeding. It depicted a gastric ulcer which was biopsied. Microscopic and microbiological analysis revealed Aspergillus spp (See Figure 2) as well as mild gastropathy changes. Special stains such as Diff-Quik stain, Alcian Blue and Periodic Acid Schiff confirmed the presence of these fungal organisms. CD4+ and CD8+ T-Lymphocytes counts are depicted in Figure 3.

The patient was started on voriconazole as first-line therapy for invasive aspergillosis [6]. No evidence of intestinal metaplasia or Helicobacter pylori was found. Unfortunately, the patient's condition continued to worsen despite appropriate therapy and the family requested withdrawal of life-support measures one day after she was diagnosed with gastric aspergillosis.

## Discussion

In patients admitted to the intensive care unit with COVID-19, opportunistic infections are common. The most prevalent opportunistic infections reported in COVID-19 patients were Aspergillus spp., Candida spp., Cryptococcus neoformans, Pneumocystis jirovecii (carinii), mucormycosis, Cytomegalovirus (CMV), herpes simplex virus (HSV), Strongyloides stercoralis, Mycobacterium tuberculosis, and Toxoplasma gondii [7].

Aspergillus spp. are the fungal microorganisms responsible for gastric aspergillosis. In most cases, inhaled fungal conidia are cleared via the mucociliary escalator in the upper airway or by alveolar macrophages. In immunosuppressed and neutropenic patients, Aspergillus fumigatus can evade the host defense mechanisms to infiltrate the vasculature’s endothelial cell lining to disseminate hematogenously to extrapulmonary sites [8]. This illness is usually found in the pulmonary parenchyma, but in rare cases, it can also be detected in the gastrointestinal tract [2]. Patients with COVID-19 have an immune response which is deregulated, leading to opportunistic agents, such as Aspergillus spp., to develop into a full infection that can have a poor outcome [3].

Conventional risk factors for opportunistic infections include chemotherapy, prolonged neutropenia, corticosteroid treatment, organ transplantation and HIV infection [9]. The increased case reports of opportunistic infections in COVID-19 raise an interest, distinctly for patients with underlying conditions who also received immunosuppressive therapy. These immunosuppressive agents utilized to reduce the inflammation are systemic corticosteroids. Even though corticosteroids are the treatment of choice for severe cases of COVID-19, these are considered immunosuppressive agents that predispose the opportunistic infections [7].

Opportunistic infections in COVID-19 patients admitted to the intensive care unit are always a possibility, due to the multiple risk factors. In our case, this pathogen was found incidentally due to a biopsy performed for a suspected upper gastrointestinal bleeding which was confirmed by a specific stain, Periodic Acid Schiff. Our patient had multiple risk factors to develop fungal infection such as prolonged invasive mechanical ventilation for nearly two weeks and glucocorticoid therapy at doses of 80mg per day to 1000 per day of methylprednisolone. After the diagnosis, the patient received doses of voriconazole of 200mg IV Q12H as recommended by the Infectious Diseases Society of America.

Although the precise epidemiological association between COVID-19 and invasive aspergillosis is unknown as there are very few reported cases [10]. It is difficult to suspect and establish an early diagnosis due to the non-specific symptoms and the capacity of the organism to be isolated from blood or tissue culture [11].

## Conclusions

SARS-CoV-2 has achieved revolutionary changes in the medical field. Over time this primary disease has been a challenge to obtain a broader understanding of all the possible secondary effects related to treatment therapy. Gastric aspergillosis can be part of the co-infections in patients with COVID-19, especially in long-term critical hospitalized patients. Patients' immunity can be compromised as part of a COVID-19 infection; as CD4 and CD8 lymphocytes decrease with the course of the infection along with steroid therapy, and in severe cases, the requirement of mechanical ventilation. This disease demands to be suspected as an uncommon presentation of opportunistic infection related to COVID-19. Gastric aspergillosis in this case was an incidental finding. Obtaining an early diagnosis is difficult due to the non-specific symptoms, nevertheless, we look forward to the evolution of treatment therapy and early diagnosis for this particular patient. This case report is helpful as it proves to be another step in the establishment of screening guidelines.
